# Evaluation of a Tobacco Treatment Training Program

**DOI:** 10.3390/ijerph19084435

**Published:** 2022-04-07

**Authors:** R. Constance Wiener, Lauren W. Swager, Melissa Suann Gaydos, Susan K. Morgan

**Affiliations:** 1 Department of Dental Public Health and Professional Practice, School of Dentistry, West Virginia University, 104a Health Sciences Addition, P.O. Box 9415, Morgantown, WV 26506, USA; rwiener2@hsc.wvu.edu (R.C.W.); mgaydos@hsc.wvu.edu (M.S.G.); 2 Department of Behavioral Medicine and Psychiatry, School of Dentistry, West Virginia University, 930 Chestnut Ridge Road, P.O. Box 9137, Morgantown, WV 26505, USA; 3 Department of Periodontics, School of Dentistry, West Virginia University, 1087 Health Sciences North, P.O. Box 9490, Morgantown, WV 26506, USA; smorgan@hsc.wvu.edu

**Keywords:** tobacco, nicotine, tobacco cessation, tobacco treatment training, TTS

## Abstract

There is a need for program evaluations associated with educating healthcare professionals about the treatment of patients who use tobacco. The purpose of this study was to evaluate a Tobacco Treatment Specialist Training program with a pre-test and post-test (provided six months after the program) to determine if participants-maintained knowledge and practices to help patients with tobacco cessation in a mixed-model analysis. A pre-test survey was administered to attendees of a three-day tobacco treatment training continuing education certification program. After 6 months, the attendees were provided a post-test survey with open-ended and Likert-style questions. There were 98 participants who completed the pre-test and 16 who completed the post-test. Responses to the knowledge, confidence, and skills post-test indicated that there was significant improvement and maintenance at the six-month post-test. For example, knowledge improved from a mean of 61.1% (SD: 25.6%) to a mean of 87.9% (SD: 14.4%); medians of 66.7% and 77.7%, respectively, *p* < 0.001. The in-depth, intensive, three-day TTS training program had a lasting impact. Providers reported greater commitment to helping their patients quit and maintain tobacco cessation habits.

## 1. Introduction

The use of tobacco is the leading preventable risk factor for premature death in Western countries [1]. While the prevalence of combustible tobacco products in the United States has declined from 42.4% in 1965 [2] to 12.5% in 2020, there were still 30.8 million adults who smoke [3]. Among youth, 1% of middle school children and 1.9% of high school children reported smoking in the past 30 days in 2021; and 2.8% of middle school children and 11.3% of high school children reported using electronic cigarettes (e-cigarettes) in the past 30 days in 2021 [3]. Among adults, e-cigarette use was 3.7% in 2020 [3]. Different formulations for tobacco and/or nicotine products are increasingly available, making people of younger or older age potentially dependent on a variety of products. Despite all efforts, tobacco use is still responsible for 20% of U.S. deaths (approximately 480,000 deaths) and shortens life expectancy by at least ten years [3].

The good news, though, is that in a recent study, 68% of people who smoke cigarettes in the U.S. want to quit [4]. In cessation research concerning e-cigarette use among youth, over half of the participants reported wanting to quit, and one-third made an attempt to quit within the previous year [5]. The researchers did not indicate success rates for the youth who made the cessation attempts. Unfortunately, less than 10% of unaided abstinence attempts result in continued success [4]. Combination counseling and evidence-based pharmacotherapy increase abstinence rates; however, less than 5% of people who want to quit receive behavioral counseling and tobacco cessation pharmacotherapy [4]. Clinicians have identified a lack of tobacco treatment training as a roadblock to providing cessation services [6].

These findings underline the need for effective interventions to assist with tobacco cessation. One powerful tool is the education of healthcare providers with evidence-based cessation strategies. This is critical as conventional products are changing and newer tobacco products are emerging.

Tobacco Treatment Specialist Training Programs (TTSTP) are educational programs for healthcare providers to have current, evidence-based knowledge and skills to encourage and assist with tobacco cessation. It was through the work of the Association for the Treatment of Tobacco Use and Dependence that the initial programs were developed and educational competencies established. Through an interprofessional effort, they formed a council (the Council for Tobacco Treatment Training Programs, or the CTTTP). In 2008 the Council developed a comprehensive approach to implementing evidence-based standards and supporting the growth of accredited programs across the U.S. Currently, there are 25 accredited programs. The goal of CTTTP is to educate healthcare participants to be able to aid people from diverse backgrounds in their tobacco cessation attempts. There are 11 competency requirements: (1) tobacco dependence knowledge and education; (2) counseling skills; (3) assessment skills; (4) treatment planning; (5) pharmacotherapy; (6) relapse prevention; (7) diversity and specific health issues; (8) documentation and evaluation; (9) professional resources; (10) laws and ethics; and (11) professional development.

The program helps healthcare to determine the intensity of treatment required to aid in tobacco cession intervention techniques. While many patients can be treated with low-intensity treatment, many individuals need high-intensity (combination) treatments to effectively address nicotine withdrawal, motivation, self-efficacy, and managing skills [7]. For this reason, an emphasis in the program is pharmacotherapy in combination with counseling as neither by itself is as effective as in combination [7]. Previous researchers indicated that healthcare providers have limited knowledge in delivering pharmacotherapy, counseling, and in providing high-intensity programs for tobacco users with high tobacco use and complex health issues [8], indicating a need for such programs. However, there is limited information about how effective tobacco cessation treatment programs are. The purpose of this study was to evaluate a TTS training program after six months to determine if participants maintain knowledge and practices to help patients with tobacco cessation.

## 2. Methods

This study received West Virginia University Institutional Board approval as an exempt protocol, number 20039360. A mixed-method study design was used.

The study sample included professional healthcare registrants, ages ≥18 years, who provided consent and completed the May 2021 TTSTP. The sample included physicians, dentists, dental hygienists, nurses, advanced practice nurses, respiratory therapists, social workers, licensed professional counselors, and addiction counselors who were licensed or students in the healthcare field.

The TTSTP was a 3-day event with multiple interprofessional speakers and participants from medicine, dentistry, social work, pharmacy, nursing, and public health. The program involved lectures about conventional and newer tobacco and nicotine delivery systems, role-playing, group discussions, treatment planning, and relapse prevention. Participants were provided information on the use of the Fagerstrom Test for Nicotine Dependence (FTND), Heaviness of Smoking Index (HS), Hooked on Nicotine Check List (HONC), Fagerstrom Nicotine Dependence Scale—Smokeless Tobacco (FTND-ST), and the Penn State Nicotine Dependence Index. The pre-test and post-test questions were developed through a consensus of experts in tobacco cessation treatment and were a sample of the most meaningful and important aspects of knowledge and practices associated with tobacco cessation.

Participation in the research study was not a requirement for participation in the educational program. Participants were informed that the questions would involve their knowledge, skills, practices, and confidence in providing tobacco cessation assistance in Likert-style questions. Open-ended questions on the post-test were used for the qualitative aspect of the study.

The research was conducted using the Kirkpatrick Four Levels for Effective Evaluation of Training programs in which (1) participant reaction, (2) learning, (3) behaviors, and (4) outcomes are considered [9]. Participant reaction (engagement/satisfaction) was examined through comments provided by the participants. The pre- and post-surveys were used to evaluate learning (knowledge). Behaviors and outcomes were also considered.

The data were analyzed with IBM^®^ SPSS^®^ Statistics Version 26 (Armonk, New York, NY, USA). Mann–Whitney U non-parametric test was used for each separate Likert-style question. A *t*-test was used for the summary knowledge scores. Significance was set at *p* < 0.05.

## 3. Results

The May 2021 TTSTP included 122 healthcare providers from medicine, dentistry, pharmacy, nursing, advanced practice nursing, respiratory therapy, licensed professional counseling, addiction counseling, and social work. Participants who responded to the pre-test were primarily (90.6%) non-Hispanic white participants, female (71.5%), and from the dental field (57.2%). Demographic data were not asked on the post-test. There were 98 (80%) participants who completed the pre-test, and 16 (13%) participants completed the post-test.

Qualitative Results

Three themes emerged from the participants’ comments. These were (1) appreciation for current, evidence-based content—foundational knowledge; (2) perception of increased confidence; and (3) increased skills in pharmacotherapy and combined treatment for tobacco cessation.

### 3.1. Theme 1: Appreciation

Common participant reactions for appreciation for having current, evidence-based content in the program included statements such as:

- “I feel like I have more complete information to offer;”
- “I will assess and advise differently;”
- “I am more knowledgeable about tobacco cessation.”
- “[I] feel more confident talking with my clients on how they can quit;”
- “[I will] provide formal tobacco cessation education; prescribe medication for tobacco cessation and perform more thorough counseling;” and
- “[I will] be doing more in prescribing; begin actively helping patients with smoking cessation more.”

### 3.2. Theme 2: Confidence

Common reactions for the second theme (increased confidence) that were evident from participants’ reactions after the program were statements such as:

- “[I] feel more confident talking with my clients on how they can quit;”
- “[I will] provide formal tobacco cessation education;
  prescribe medication for tobacco cessation;
  and perform more thorough counseling;” and
- “[I will] be more confident in prescribing;
  begin actively helping patients with smoking cessation more.”

### 3.3. Theme 3: Skills

The third theme centered around the participants’ recognition of having increased skills in pharmacotherapy and combined treatment for tobacco cessation. This then was exemplified by statements such as:

- “I can now apply evidenced based medicine and safely and efficiently prescribe, to best serve my patients and community;”
- “Offer pharmacotherapy;” and
- “Recommend combination pharmatherapy [sic].”

Quantitative Results

For the nine learning/knowledge questions, the mean score on the pre-test was 61.1% (SD: 25.6%), and the median was 66.7% correct. The mean score on the post-test was 87.9% (SD: 14.4%), and the median was 77.8%, *p* < 0.001. Details for specific questions are presented in Table 1.

Table 2 includes the behavioral/outcome comparisons of confidence levels in the skills associated with providing tobacco cessation information, treatment, and support to patients. These included asking about tobacco use, advising quitting, assessing quitting willingness, assisting with quitting, indicating appropriate nicotine replacement treatment to consider, and prescribing a combination therapy. Initially, and at 6 months, the participants were just as likely to ask about tobacco use. Furthermore, all other levels of confidence with these questions improved from the pre-test to the post-test.

Table 3 includes the comparison of the level of skill from the participant’s self-perception of having anywhere from a novice to expert level in their understanding of behavior/outcome. Participants at 6 months were more likely to report “expert” levels of skill in communicating health consequences of tobacco, documenting cessation progress in patient charts, determining individual needs for cessation treatment options, identifying patients who needed medical referrals for cessation, and identifying patients who needed psychological referrals for cessation.

Table 4 includes the responses to current behavioral/outcome practices. The recommendation of nicotine patches was significantly different between the pre-test and post-test, with participants responding more positively to the post-test. There were no significant differences among participants from the pre-survey to the post-survey in asking permission to provide tobacco cessation advice and the utilization of reflections or open-ended questions when counseling patients about tobacco cessation.

## 4. Discussion

In this study, participants reported significant improvement in knowledge, confidence, and evidence-based practices in managing patients with tobacco cessation on a post-test administered six months following a TTSTP program. Participants were more likely to report being at an expert level in skills associated with tobacco cessation on the post-test as compared with the pre-test. Comments about the skills learned and confidence gained were positive, and the participants expressed appreciation for having had the learning experience.

There is a lack of comparable program evaluation studies related to tobacco cessation programs for healthcare provider interprofessional education. In one similar study of program evaluation, the researcher evaluated a train-the-trainer program [10]. However, that program was web-based, while the current study was in person. The researcher found the program to be effective for increasing confidence in the pharmacy faculty, and intention to continue to use the curriculum in their courses [10]. The researcher also reported the program to be effective among participants who provided patient care [10]. Most participants reported that they asked about tobacco use all or almost all the time; most used motivational interviewing/integrated brief counseling; half provided Quitline cards/numbers, and 41.2% checked for potential smoking-drug interactions when filling prescriptions [10]. Our study showed participants reported similar results on the post-test.

In another study, researchers used a pre-test/end-of-academic year post-test format to evaluate a 4-h webinar format, an interprofessional faculty train-the-trainer program designed for respiratory therapy faculty [11]. The emphasis of their program was the preparation of faculty in teaching tobacco cessation. Although our program had many participants who were faculty members and planned to present tobacco cessation information in their classes, the intent of our research was to educate healthcare providers to be competent in providing tobacco cessation treatments for their patients. Our program also differed in length and presentation mode; however, both programs had similar topics for foundational knowledge and patient counseling.

Another tobacco evaluation study was conducted with qualitative methods. The researchers interviewed 18 participants of the Rx for Change: Clinician-Assisted Tobacco Cessation program and reported that participants had increased confidence and counseling skills, enhanced treatment practices, and useful background information even 12–14 years after having participated in the program [12]. Similar themes were evident in our research. One theme was an appreciation for current, evidence-based content/foundational knowledge, which parallels the theme of useful background information in the Rx for Change study. Our theme of the perception of increased confidence is similar to theirs of increased confidence and counseling skills. Furthermore, our third theme of increased skills in pharmacotherapy and combined treatment for tobacco cessation was similar to theirs of enhanced treatment practices. Our study was also similar to theirs in that both were nationally recognized programs, and both have similar long-term effects. Our study did differ in that we had (1) an interprofessional faculty, (2) interprofessional healthcare participants, (3) an emphasis on established healthcare providers, and (4) our post-survey was distributed 6 months after the program.

In a study in which researchers used a data-driven community-based participatory research approach, online educational modules were created and tested by community leaders and academic researchers [13]. They conceptualized and developed a curriculum that was culturally specific and met components that addressed ethnic and dispositional community characteristics. Then the researchers conducted a summative evaluation through semi-structured interviews and feedback sessions. Much of the research included qualitative outcomes, which served as the basis for trial and error, flexible planning, and continuous improvement. The current study had a similar approach in which many presenters gathered to develop and present the curriculum to meet the needs of the various healthcare providers. The program was adjusted for responding to participant needs, and lessons were learned in providing a curriculum that had lasting effects.

### 4.1. Strengths and Limitations

This study has both strengths and limitations. A study limitation is the number of responses received on the post-test. It was sent to participants twice. However, the response rate was low. Reasons for the low response rate include the impact of the COVID-19 pandemic on available healthcare provider time and changes in contact information over the six months of the study. A strength of the study is the use of questions that had been vetted by experts in tobacco treatment to hone in upon the factor that was most important: helping patients to quit tobacco use and maintain being tobacco-free.

### 4.2. Future Lines of Action

There remains a considerable need to help patients with tobacco cessation. Programs that have summative success, such as this program, are of great importance to public health. They should be supported and expanded into professional healthcare students’ education. Future research should include economic impact based on the number of successful tobacco cessations participants experience after having completed the program.

## 5. Conclusions

While there was a limited six-month response rate in this study, results showed overwhelming improvement in knowledge, confidence, and skills in managing patients with tobacco cessation. The in-depth, intensive, three-day TTS training program had a lasting impact on those responding healthcare providers’, and some rated themselves at the expert level in many skill areas associated with cessation. More research needs to be completed on the impact of TTS training, particularly as conventional and newer tobacco products continue to flood the market.

## Figures and Tables

**Table 1:** Learning/Knowledge Gained between the Pre-test and Post-test.

	Correct	Strongly						Strongly				
Item	Response	Disagree		Disagree		Neutral		Agree		Agree		*p-*Value
One typical cigarette has 1–1.5 mg nicotine	TRUE											**0.036**
Pre-test		15	15.50%	12	12.40%	21	21.60%	39	40.20%	10	10.30%	
Post-test		0		1	6.30%	1	6.30%	3	18.80%	11	68.80%	
One pack of cigarettes typically has 50 cigarettes	FALSE											**0.036**
Pre-test		60	61.90%	21	21.60%	7	7.20%	8	8.20%	1	1.00%	
Post-test		14	87.50%	2	12.50%	0		0		0		
If a person smokes 2 packs of cigarettes a day, according to the Mayo Clinic, it is OK to use two 21 mg nicotine patches at the same time	TRUE											**<0.001**
Pre-test		15	15.50%	24	24.70%	25	25.80%	25	25.80%	8	8.20%	
Post-test		0		1	6.30%	1	12.50%	7	43.80%	6	37.50%	
Bupropion SR (Zyban^®^) should NOT be used in people with liver diseases or prone to seizures	TRUE											**0.021**
Pre-test		1	1.00%	2	2.10%	28	28.90%	50	51.50%	16	16.50%	
Post-test		0		1	6.30%	1	6.30%	7	43.80%	7	43.80%	
The nicotine in one can of some brands of snuff is equal to 4 packs of cigarettes	TRUE											**<0.001**
Pre-test		0		5	5.20%	33	34.40%	49	51.00%	9	9.40%	
Post-test		0		0		0		10	62.50%	6	37.50%	
For Bupropion SR (Zyban^®^) use, a person is still smoking in days 1–7; 150 mg Bupropion is taken once a day in days 1–3; and twice a day (8 h apart) in days 4–7. The person stops smoking in day 8.	TRUE											0.175
Pre-test		4	4.20%	7	7.40%	44	46.30%	37	38.90%	3	3.20%	
Post-test		1	6.30%	2	12.50%	4	25.00%	4	25.00%	5	32.30%	
Pack-years is the number of packs of cigarettes smoked per day time the number of years a person smoked	TRUE											**0.007**
Pre-test		1	1.00%	3	3.10%	10	20.80%	46	47.90%	26	27.10%	
Post-test		0		1	6.30%	0		5	31.30%	10	62.50%	
Evidence-based information supports the												
Safety of e-cigarettes	FALSE											0.373
Pre-test		49	52.70%	30	32.30%	13	14.00%	1	1.10%	0		
Post-test		10	62.50%	5	31.30%	1	6.30%	0		0		
An individual with tobacco use disorder’s ambivalence toward smoking is addressed in motivational interviewing	TRUE											**0.007**
Pre-test		1	1.10%	1	1.10%	39	41.50%	42	44.70%	11	11.70%	
Post-test		0		1	6.30%	2	12.50%	5	31.30%	8	50.00%	
My team provides a consistent message toward tobacco cessation												**0.031**
Pre-test		1	1.10%	7	7.40%	29	30.50%	42	44.20%	16	16.80%	
Post-test		0		0		2	13.30%	8	53.30%	5	33.30%	
Mean scores for questions 1–9												**<0.001**
Pre-test	5.5 (Standard deviation: 2.3)			61.10% (SD: 25.6%)		Median 6		66.70%				
Post-test	7.9 (Standard deviation: 1.3)			87.80% (SD: 14.4%)		Median 7		77.70%				

Boldface indicates statistical significance (*p* < 0.05) using Mann-Whitney U Test for each question and *t*-test for the summary scores based on 98 pre-test responses and 16 post-test responses for the total scores.

**Table 2:** Behavioral/outcome practices: Level of Confidence Gained between the Pre-test and Post-test.

	Very Low		Low		Neutral		High		Very High				
Item	Confidence		Confidence		Confidence		Confidence		Confidence		N/A		*p-*Value
Asking about a person’s tobacco use													0.055
Pre-test	1	1.00%	2	2.10%	13	13.50%	40	41.70%	34	35.40%	6	6.30%	
Post-test	0		0		0		5	31.30%	11	68.80%	0		
Advising patients who use tobacco to quit													**0.007**
Pre-test	3	3.10%	8	8.30%	30	31.30%	29	30.20%	20	20.80%	6	6.30%	
Post-test	0		0		1	6.30%	6	37.50%	9	56.30%	0		
Providing tobacco cessation assessment													**<0.001**
Pre-test	3	3.10%	22	22.90%	38	39.60%	20	20.80%	7	7.30%	6	6.30%	
Post-test	0		0		1	6.30%	9	56.30%	5	31.30%	1	6.30%	
Assisting a patient to quit tobacco use													**<0.001**
Pre-test	5	5.30%	20	21.1%	29	30.50%	26	27.40%	9	9.50%	6	6.30%	
Post-test	0		0		1	6.30%	7	43.80%	7	43.80%	1	6.30%	
Arranging a tobacco cessation program for a patient													**<0.001**
Pre-test	12	12.50%	25	26.00%	29	30.20%	18	18.80%	6	6.30%	6	6.30%	
Post-test	0	0	4	25.00%	8	50.00%	3	18.80%	1	6.30%	0		
Telling patients the appropriate manner or NRT use													**0.001**
Pre-test	12	12.50%	25	26.00%	24	25.00%	24	25.00%	4	4.20%	7	7.30%	
Post-test	0		1	6.30%	2	12.50%	8	50.00%	4	25.00%	1	6.30%	
Prescribing combination therapy													**0.011**
Pre-test	20	20.80%	17	17.70%	24	25.00%	13	13.50%	1	1.00%	21	21.90%	
Post-test	0		2	12.50%	1	6.30%	7	43.80%	2	12.50%	4	25.00%	

Boldface indicates statistical significance (*p* < 0.05) using Mann-Whitney U Test for each question based on 98 pre-test responses and 16 post-test responses.

**Table 3:** Behavioral/outcome practices: Self-Perceived Level of Skill Comparison of Pre-test and Post-test.

Self-Perception													
Item	Novice		Learner		Neutral		Competent		Expert		N/A		*p-*Value
Communicating health consequences of tobacco													**<0.001**
Pre-test	2	2.10%	15	15.60%	22	22.90%	51	53.10%	5	5.20%	1	1.00%	
Post-test	0		0		0		12	75.00%	4	25.00%	0	0	
Documenting tobacco cessation progress in patient charts													**0.002**
Pre-test	16	16.80%	15	15.80%	29	30.50%	25	26.30%	1	1.10%	9	9.50%	
Post-test	0		1	6.30%	3	18.80%	6	37.50%	4	25.00%	2	12.50%	
Determining individual-specific appropriate tobacco cessation options													**<0.001**
Pre-test	15	15.80%	28	29.50%	30	31.60%	17	17.90%	0		5	5.30%	
Post-test	0		1	6.30%	2	12.50%	9	56.30%	4	25.00%	0		
Identifying risk factors requiring medical referrals for tobacco cessation													**0.004**
Pre-test	15	15.90%	28	29.50%	30	31.60%	17	17.90%	0		5	5.30%	
Post-test	0		1	6.30%	2	12.50%	9	56.30%	4	25.00%	0		
Identifying risk factors requiring referrals for psychological help with tobacco cessation													**0.003**
Pre-test	16	16.80%	21	22.10%	32	33.70%	21	22.10%	0		5	5.30%	
Post-test	0		2	12.50%	3	18.80%	9	56.30%	2	12.50%	0		

Boldface indicates statistical significance (*p* < 0.05) using Mann-Whitney U Test for each question based on 98 pre-test responses and 16 post-test responses.

**Table 4:** Behavioral/outcome practices: Current Practice Changes Between the Pre-test and Post-test.

	Very						Very						
Item	Low		Low		Neutral		High		High		N/A		*p-*Value
I ask permission to provide tobacco cessation advice													0.313
Pre-test	2	2.10%	7	7.30%	28	29.20%	33	34.40%	14	14.60%	12	12.50%	
Post-test	0		0		3	18.80%	9	56.30%	2	12.50%	2	12.50%	
I utilize reflective listening when counseling patients													0.433
Pre-test	1	1.00%	0		24	25.00%	45	46.90%	17	17.70%	9	9.40%	
Post-test	0		1	5.30%	2	12.50%	7	43.80%	4	25.00%	2	12.50%	
I use open-ended questions when counseling patients													0.257
Pre-test	1	1.10%	2	2.10%	21	22.30%	44	46.80%	17	18.10%	9	9.60%	
Post-test	0		0		3	18.80%	6	37.50%	5	31.30%	2	12.50%	
I recommend nicotine patches for tobacco cessation													**0.007**
Pre-test	6	6.30%	5	5.20%	37	38.50%	26	27.10%	6	6.30%	16	16.70%	
Post-test	0		0		2	12.50%	5	31.30%	7	43.80%	2	12.50%	

Boldface indicates statistical significance (*p* < 0.05) using Mann–Whitney U test for each question based on 98 pre-test responses and 16 post-test responses.

## Data Availability

Data sharing is not applicable to this article.
